# Reward Deficiency Syndrome: Attentional/Arousal Subtypes, Limitations of Current Diagnostic Nosology, and Future Research

**DOI:** 10.17756/jrds.2015-002

**Published:** 2015-02-06

**Authors:** Edward Justin Modestino, Kenneth Blum, Marlene Oscar-Berman, Mark S. Gold, Drake D. Duane, Sarah G.S. Sultan, Sanford H. Auerbach

**Affiliations:** 1 Department of Neurology, Boston University School of Medicine, Boston, MA, USA; 2 Department of Neurology, Boston VA Healthcare System, Boston, MA, USA; 3 Department of Psychiatry & McKnight Brain Institute, University of Florida College of Medicine, Gainesville, FL,USA; 4 Human Integrated Services Unit University of Vermont Center for Clinical & Translational Science, Department of Psychiatry, College of Medicine, Burlington, VT, USA; 5 Dominion Diagnostics, LLC, North Kingstown, RI, USA; 6 Department of Addiction Research & Therapy, Malibu Beach Recovery Center, Malibu Beach, CA, USA; 7 Departments of Psychiatry, Anatomy & Neurobiology, Boston University School of Medicine, Boston, MA, USA; 8 *Boston VA Healthcare System, Boston, MA, USA*; 9 Departments of Psychiatry & Behavioral Sciences at the Keck, University of Southern California, School of Medicine, CA, USA; 10 Director of Research, Drug Enforcement Administration (DEA) Educational Foundation, Washington, D.C, USA; 11 Institute for Developmental Behavioral Neurology Scottsdale, AZ, USA; 12 Department of Speech and Hearing Sciences, Arizona State University, Tempe, AZ, USA; 13 Department of Neurology, The University of Arizona College of Medicine-Phoenix, Phoenix, AZ, USA; 14 Department of Psychiatry, St. Mary's Hospital Centre, Montreal, QC, Canada; 15 Department of Psychiatry, Faculty of Medicine, McGill University, Montreal, QC, Canada; 16 Departments of Neurology & Psychiatry, Boston University School of Medicine, Boston, MA, USA; 17 Sleep Disorders Center & Behavioral Neurology, Boston Medical Center, Boston, MA, USA

**Keywords:** ADHD, Narcolepsy, Reward deficiency syndrome, Genetics, fcMRI

## Abstract

We theorise that in some cases Attention Deficit Hyperactivity Disorder (ADHD) predisposes to narcolepsy and hypersomnia, and that there may be a shared pathophysiology with various addictions [Reward Deficiency Syndrome (RDS)]. Reticence to acknowledge such connections may be due to a narrow nosological framework. Additionally, we theorise that the development of narcolepsy on a baseline of ADHD/RDS leads to an additional assault on the dopaminergic reward system in such individuals. In this study, we propose to test these hypotheses by using a combination of broad genetic screening, and neuroimaging with and without pharmacological intervention, in those with pure ADHD, pure narcolepsy, and the combined ADHD-narcolepsy phenotype. Results of this proposed study may reveal a common pathophysiology of ADHD, narcolepsy and RDS, and perhaps an additional compromise to the reward system in those with combined ADHD-narcolepsy. If the evidence supports the hypothesis that indeed there is a shared pathophysiology for narcolepsy with RDS and thus its subtype ADHD, early intervention/preventative treatment amongst those with ADHD may be beneficial with the putative dopaminergic compound KB220Z™.

In a recently published study, Modestino and Winchester [1] provided evidence of a significant retrospective childhood history of Attention Deficit Hyperactivity Disorder (ADHD) symptomatology among adult narcoleptics. The authors suggested that a subtype of ADHD may be the early stages in a progressive neurological syndrome that ultimately manifests in hypersomnias. In addition to their findings, the foundation for that study was supported by results of previous research. Yoss [2] was able to show the same objective pupilographic instabilities associated with hypo-vigilance in those with ADHD (then known as minimal brain dysfunction) and narcolepsy. Duane [3] continued this work with pupilography and stated that 50 per cent of his patients with ADHD were hypo-vigilant. Weinberg and Harper [4] observed a group of individuals (six case studies) with all three core symptoms of ADHD (hyperactivity, impulsivity, and inattentiveness) who also had daytime sleepiness and sleep attacks (some of whom had comorbid diagnoses of ADHD and narcolepsy). Weinberg termed this *Primary Disorder of Vigilance*. Sultan and associates [5] identified a group of individuals with a narcolepsy spectrum disorder, *Syndrome Z*, which included a DSM-IV diagnosis of ADHD, REM (rapid eye movement) sleep behaviour disorder, excessive daytime sleepiness, cataplexy, and many were HLA-DR2 positive. Most recently, Ohayon [6] showed that significantly (p<.01) more narcoleptics (5.4%) than controls (2.5%) were diagnosed with ADHD in childhood. In addition to finding significance with comparison of a control population, if Ohayon had performed a joint preference calculation (refer to Modestino and Winchester [1], for an explanation of this calculation) to rule out random co-occurrence of ADHD and narcolepsy, he would have discovered that far less than one percent of his narcoleptics should have had a childhood diagnosis of ADHD. Therefore, he had greater than five times the joint prevalence that would be present if there were no authentic association, or mere chance/coincidence, between childhood ADHD and the subsequent development of narcolepsy in his population of narcoleptics.

Debate and scepticism have surrounded the notion of a connection between ADHD and hypersomnias. Oosterloo and colleagues [7] claimed that the diagnostic confusion is due to the overlap in symptoms observed between the two disorders. Narcolepsy and hypersomnia can cause ADHD-like symptoms, and ADHD, especially the inattentive subtype, can resemble hypersomnias. Despite this, there is no firm overlap in the diagnostic criteria of symptoms between ADHD and hypersomnias, making it appear that they are easily distinguishable from one another. Perhaps this reticence to see an authentic connection between ADHD and narcolepsy may be due to a narrow diagnostic mind-set, and the dichotomy of the historical stereotypes of ADHD (i.e., hyperactive children) [8] and narcolepsy (i.e., sleepy adults) [9].

An alternative framework to this issue has been offered, which questions the limitations of strict diagnostic nosology, where many patients have atypical syndromes combined with a blur of multifarious comorbidities. Antidepressant response, in addition to common comorbidities, has revealed a cluster of disorders [major depressive disorder, dysthymic disorder, ADHD, cataplexy (a component of narcolepsy), bulimia nervosa, obsessive-compulsive disorder, social phobia, panic disorder, generalised anxiety disorder, post-traumatic stress disorder, premenstrual dysphoric disorder, migraine, fibromyalgia, and irritable bowel syndrome] that may share a common pathophysiology and perhaps genes suggestive of pleiotropy. This cluster of disorders was titled Affective Spectrum Disorder (ASD), and interestingly includes both ADHD and narcolepsy [10, 11]. Following in the steps of ASD, a more recent study demonstrated that single-nucleotide polymorphisms at four loci (calcium ion channel regulating genes) are shared with five pathologies (autism spectrum disorder, ADHD, bipolar disorder, major depressive disorder, and schizophrenia). This study provides evidence for pleiotropy amongst these five pathologies. The authors suggested that nosology should be based on cause of disease, not by symptoms alone [12]. Along this same line of thinking, due to heterogeneity within diagnoses, and frequent comorbidities, a large scale initiative in the U.S. initiated by the National Institute of Mental Health, titled the Research Domain Criteria (RDoC) project, has pushed scientists and researchers to rethink traditional symptom based nosology and narrow diagnostic categorisation. This new methodology examines the interface of behavioural symptoms, cognitive styles, various neurobiological measures, and genetics; all in the expectation of understanding brain disorders and related pathologies more holistically [13, 14].

In this same vein of nosological integration, there is Reward Deficiency Syndrome (RDS). RDS is a cluster of syndromes across addictive, compulsive, and impulsive spectrums [substance use disorder (including drug addiction, alcoholism, and smoking), eating disorders, sexual addictions, gambling, compulsive shopping, risk taking behaviour, anxiety, irritability, and ADHD] where there is a dysregulation of dopamine that makes an impact on the reward system. Remarkably, through innervation and various neural cascades, RDS encompasses various pathologies across multiple neurotransmitter systems, which ultimately lead to a dysregulation of this same dopaminergic reward system [15-17]. For more details of this intricate cascade, please refer to Blum et al. [16] and Figure 1. In Figure 1, pathways 1a., 2., 3., and 4. are pathways associated with RDS (which includes ADHD). Based on evidence from Calipari and España [18], we have added in pathway 1b. into Figure 1: hypocretin/orexin excitatory modulation of dopamine in the ventral tegmental area (directly *via* receptors on dopaminergic neurons, and indirectly by augmenting glutamatergic excitability of dopaminergic neurons via increasing NMDA receptor number) to this cascade. This 1b. pathway is associated with narcolepsy.

Despite the tight linkage with HLA-DQB1*0602, narcolepsy appears to be polygenic, and thus associated with the interaction of various genes and environmental factors [19, 20]. One of the various genes associated with RDS and ADHD is the ***Taq A1*** allele variant of the D2 dopamine receptor, in addition to genes for COMT, GABA, serotonin, etc. [15-17]. Interestingly, a variant of the D2 allele has been associated with narcolepsy as well, in addition to genes for COMT, GABA, serotonin, etc. [20]. Thus, in line with the evidence of polygenic pathologies, including the issues of pleiotropy [12], and RDoC [13, 14], we suggest a connectomic approach, combining neuroimaging and genetic screening such as that used by Fornito and Bullmore [21], to further examine RDS and its potential hypersomnia subtypes. We theorise that hypersomnias, including narcolepsy, which may emerge from a background history of ADHD [22, 23, 1, 6], may also fit under the umbrella of RDS. Furthermore, we suggest that the initial dopaminergic deficiency seen in ADHD/RDS is markedly worsened with the onset of narcolepsy. More specifically, we propose to examine the resting-state functional connectivity magnetic resonance imaging (rs-fcMRI) of reward circuitry (as previously studied in children with ADHD by Costa Dias et al.) [24] in those with adult ADHD (with no history or symptoms of hypersomnias), those with narcolepsy (with no history or historical/documented symptoms of ADHD prior to the emergence of the sleep disorder), and those with combined ADHD-narcolepsy (with an authentically documented lifelong history of ADHD prior to the development of the full-blown sleep disorder), both on and off a putative dopaminergic compound KB220Z™, in conjunction with a genetic screening. This will allow us to tease apart shared and unique genetic and brain connectivity patterns within these potential RDS subtypes.

KB220Z™ is a complex that has been extensively studied in pre-clinical and human trials [25]. Note: this compound follows the cascade shown in Figure 1, increasing dopamine production and release directly and indirectly through other systems via innervation. As reported in a detailed review article [25] on both animals and humans to date, KB220 variants have been shown to enhance brain enkephalin levels in rodents; reduce alcohol-seeking behaviour in C57/BL mice; pharmacogenetically convert ethanol acceptance in preferring mice to non-preferring mice, such as DBA/2J. In humans, KB220Z™ has been reported to reduce drug and alcohol withdrawal symptomatology (i.e. lower need for benzodiazepines, reduced days with withdrawal tremors, evidence of a lower BUD score [building up to drink], and no severe depression on the MMPI. Patients in recovery treatment had reduced stress response, as measured by the skin conductance level (SCL), and significantly improved Physical Scores and BESS Scores (behavioural, emotional, social and spiritual). After detoxification there was a six-fold decrease in Against Medical Advice (AMA) rates when comparing KB220 variant to placebo groups. Healthy volunteers demonstrated an enhanced focus. There is also evidence of reduced craving for alcohol, heroin, cocaine, nicotine. Also, reductions in inappropriate sexual behaviour and reduced post-traumatic stress (PTSD) symptoms, such as lucid nightmares, have been reported [26]. Quantitative electroencephalic (qEEG) studies in humans have found that KB220Z™ modulates theta power in anterior cingulate cortex. In abstinent heroin addicts a single dose of KB220Z™ compared to placebo in a pilot study [25, 27] resulted in activation of the nucleus accumbens as well as activation and improvement of the prefrontal-cerebellar-occipital neural network using rs-fcMRI. In addition it was found that carriers with the DRD2 A1 allele showed a significant Pearson correlation in terms of enhanced compliance to KB220Z™ treatment relative to carriers of the normal compliment of DRD2 receptors in known obese patients [28]. This further suggests the importance of low dopamine function equates to better treatment outcome.

Prior to commencing the study, all research will be approved by the local ethics committee/institutional review boards in accordance with the ethical standards of the 1964 Declaration of Helsinki. Furthermore, informed consent will be obtained from all participants. Diagnoses of ADHD, narcolepsy, and the combined phenotype will be confirmed by a clinical interview with a licensed clinician, in addition to medical records.

## Figures and Tables

**Figure 1:**
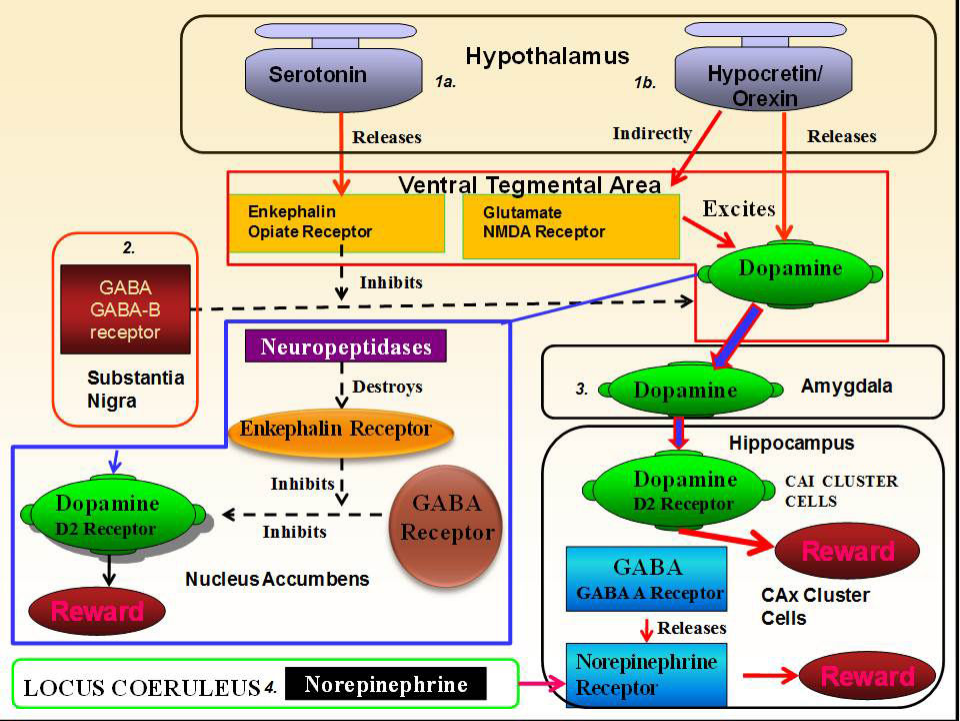
Interactions in brain reward regions associated with RDS [Adapted from Blum et al. (2008)] [16] (1a.) Hypothalamus: Serotonin indirectly activates opiate receptors, causing a release of enkephalins in the ventral tegmental area (VTA). The enkephalins inhibit the firing of GABA, which originates in the substantia nigra. (2.) GABA: GABA acts through GABA B receptors to inhibit/regulate release of dopamine (DA) at the VTA, projecting to nucleus accumbens (NAcc). Release of DA in NAcc activates DA D2 receptors. This same release is modulated by enkephalins via GABA. Enkephalins are regulated by neuropeptidases. (3.) DA also is released in the amygdala. Projecting from the amygdala, DA stimulates the hippocampus (HIPP) where CA cells excite DA D2 receptors. (4.) Additionally, norepinephrine (NE) in the locus coeruleus innervates the HIPP around CAx (a cluster of cells yet to be identified). In the HIPP, excitation of GABA A receptors causes the release of NE. (1b.) Hypothalamus: Hypocretin/orexin release from the lateral hypothalamus causes an excitatory modulation of DA in the VTA (directly via receptors on dopaminergic neurons, and indirectly by augmenting glutamatergic excitability of dopaminergic neurons via increasing NMDA receptor number).
